# Sensitivity and responsiveness of the EQ-5D-3L in patients with uncontrolled focal seizures: an analysis of Phase III trials of adjunctive brivaracetam

**DOI:** 10.1007/s11136-016-1483-3

**Published:** 2016-12-21

**Authors:** Clara Mukuria, Tracey Young, Anju Keetharuth, Simon Borghs, John Brazier

**Affiliations:** 1 School of Health and Related Research, University of Sheffield, Regent Court, 30 Regent Street, Sheffield, S1 4DA UK; 2 UCB Pharma, Slough, UK

**Keywords:** EQ-5D-3L, Uncontrolled focal seizures, QOLIE-31P, Validity, Quality of life

## Abstract

**Purpose:**

Preference-based measures are required to measure the impact of interventions for cost-effectiveness analysis. This study assessed the psychometric performance of the EQ-5D-3L in adults with uncontrolled focal (partial-onset) seizures.

**Methods:**

Data from three Phase III studies of an antiepileptic drug (adjunctive brivaracetam; *n* = 1095) were used. Analysis included correlations between EQ-5D-3L and Quality of Life in Epilepsy Inventory (QOLIE-31P) and seizure frequency. Known group validity was based on ability of the EQ-5D-3L to discriminate between baseline QOLIE-31P total scores, seizure type and number of antiepileptic drugs using effect sizes (ES). Responsiveness assessed proportions reporting highest or lowest scores, overall change using standardized response means (SRM) and change by responder and clinician/patient evaluation groups using ES.

**Results:**

Correlations were weak to moderate (*ρ* = 0.2–0.4) between EQ-5D-3L dimensions and QOLIE-31P subscales, apart from medication effects (*ρ* < 0.1); seizure frequency was not associated with either measure. Known group analysis had small ES. A quarter (24.9%) of patients had a baseline EQ-5D-3L utility score of 1 (full health) but lower average QOLIE-31P scores. SRMs were small (<0.1) in EQ-5D-3L compared with 0.1–0.4 for QOLIE-31P subscales. Results across the studies were mixed for responder status and clinician/patient evaluation of improvement for EQ-5D-3L.

**Conclusions:**

EQ-5D-3L had weak-to-moderate correlations with QOLIE-31P and varied with QOLIE-31P severity groups, but showed less responsiveness than QOLIE-31P. Given this lack of sensitivity, EQ-5D-3L may not be appropriate for measuring the impact of interventions in cost-effectiveness analysis in this population and disease-specific preference-based measures may be more appropriate.

**Electronic supplementary material:**

The online version of this article (doi:10.1007/s11136-016-1483-3) contains supplementary material, which is available to authorized users.

## Introduction

Epilepsy is a heterogeneous group of central nervous system disorders characterized by unpredictable recurrent seizures [1]. Epilepsy can significantly affect patients’ health-related quality of life (HRQoL), including their mental health, and role and social functioning [2]. Seizure control can be achieved with antiepileptic drug (AED) treatment [3, 4], but up to 30% of patients still have uncontrolled seizures. HRQoL can also be affected by AED-associated side effects even in controlled patients. HRQoL measures used in epilepsy trials should capture these varied effects.

Reimbursement agencies such as the National Institute for Health and Care Excellence (NICE) in the UK require effectiveness to be measured in terms of quality-adjusted life years (QALYs) [5], which combines HRQoL with length of life. The quality of life component of QALYs can be estimated using generic preference-based measures, such as the EQ-5D-3L [6] and the Short Form 6 dimensions (SF-6D) [7] which are recommended by some reimbursement agencies such as NICE. EQ-5D-3L is the most widely used generic preference-based measure. Generic preference-based measures enable comparison across different diseases and populations and, as such, ensure a consistent basis for the assessment of cost-effectiveness. Alternatively, disease-specific preference-based measures can be used to generate QALYs, though these are not comparable with QALY estimates derived from other instruments. Where preference-based measures have not been used in clinical trials, some reimbursement agencies such as NICE allow mapping of data from disease-specific *non*-preference-based measures, such as the epilepsy-specific Quality of Life in Epilepsy Inventory (QOLIE-31P) [8], to generic preference-based measures, for use in cost-effectiveness analysis [5].

In order to generate robust cost-effectiveness data, generic preference-based measures need to be validated in the population of interest; this also applies when mapping is to be used [9]. However, few mapping studies report information on validity of the generic preference-based measures used in their analysis. There is evidence that generic preference-based measures do not adequately cover dimensions of HRQoL affected by certain diseases, including epilepsy, and as such are not usable. This coverage issue was found in a study (*n* = 140) of patients evaluated for epilepsy surgery [10]. Therefore, the primary aim of this study was to examine the validity and responsiveness of the EQ-5D-3L in a large sample of patients with epilepsy who had uncontrolled focal (partial-onset) seizures and who were taking part in trials of an approved antiepileptic drug. A secondary aim was to test mapping from the QOLIE-31P to the EQ-5D-3L if the two measures were shown to have sufficient overlap based on the psychometric performance of EQ-5D-3L, although there are no specific guidelines in the literature regarding what is acceptable for mapping purposes [9].

## Methods

### Data sources

Analyses used data from three double-blind, randomized, placebo-controlled, Phase III studies of adjunctive brivaracetam, which is approved as an adjunctive therapy for focal seizures, in adults with uncontrolled focal seizures: N01252 (NCT00490035) [11]; N01253 (NCT00464269) [12]; N01254 (NCT00504881) [13]. The study designs have previously been described in the literature [11–13]. Baseline characteristics of the patients revealed the refractory nature of their epilepsy. Most patients had previously taken several other AEDs: 50.3% had 2–4 prior AEDs, 14.8% had ≥5 prior AEDs, and 66.1% were taking two concomitant AEDs. Mean time since diagnosis was 20.4–26.2 years, and patients reported a median of 1.8–2.9 focal seizures/week at baseline. All three studies used the EQ-5D-3L and QOLIE-31P to measure HRQoL.

### Measures and assessments

#### EQ-5D-3L

The EQ-5D-3L is a preference-based measure of health status across five dimensions (mobility; self-care; usual activities; pain/discomfort; and anxiety/depression), each with three severity levels (no problems, some problems, and extreme problems). This results in a total of 243 possible ‘health states’, i.e. combinations of dimensions and severity levels. A subset of the health states has been evaluated in the general population and modelled to provide the utility tariff for each health state, where 1 represents full health, 0 represents dead and scores below 0 ‘worse than dead’. In this analysis, the UK tariff was applied, which ranges from −0.594 to 1 [14].

#### QOLIE-31P

The QOLIE-31P is a non-preference-based questionnaire adapted from the QOLIE-31 which includes 30 items grouped into seven subscales: energy/fatigue (4 items); emotional well-being (5 items); daily activities/social functioning (5 items); cognitive functioning (6 items); medication effects (3 items); seizure worry (5 items); overall quality of life (2 items); and a health status item. It also includes 7 items on the degree of ‘distress’ for each subscale topic and a ‘prioritization’ item on the relative importance of each subscale topic. The total score is a weighted sum of the subscale scores. Scores range from 0 to 100, with higher scores representing better functioning [15].

Patients without mental impairment (as judged by the investigator) in the three brivaracetam trials self-completed the EQ-5D-3L and QOLIE-31P questionnaires at baseline and at weeks 4, 8 and 12 (plus week 16 in N01254). Data analysed were from baseline and week 12 (week 16 for N01254).

#### Seizure frequency, severity and type

Patients completed daily seizure record cards, including date, time, frequency, type of seizures and occurrence of seizure clusters. Record cards were reviewed at visits to ensure completeness and accuracy. Focal seizures were classified as simple, complex or secondarily generalized [16]. The presence of the latter was used in this analysis as a proxy for disease severity.

#### Number of prior AEDs

The number of different AEDs used in the past 5 years was recorded at baseline. Patients with more prior AEDs could be considered more treatment refractory, which in turn could be related to baseline HRQoL.

#### Patient’s and Clinician’s Global Evaluation Scales (PGES/CGES)

At weeks 12 or 16, patients without mental impairment completed the patient global evaluation scale (PGES). Investigators completed the clinician global evaluation scale (CGES). Patients were asked ‘Overall, has there been a change in your seizures since the start of the study medication?’. Investigators were asked to ‘Assess the overall change in the severity of the patient’s illness, compared with the start of study medication’. Response options ranged from ‘marked improvement’ (7) to ‘marked worsening’ (1), with a score of 4 representing ‘no change’. In all three trials, very few respondents reported marked (1), moderate (2) or slight worsening (3) in either PGES (≤7%) or CGES (≤4%); these options were therefore combined with the ‘no change’ category in this analysis.

### Analysis

#### Construct validity

Construct validity measures how well an instrument assesses what it was intended to assess. Assessment of construct validity relies on techniques that provide information on whether the instrument is related to or converges with other measures that cover constructs of interest, referred to as convergent validity, as well as whether an instrument is able to distinguish between groups that have known differences (known group validity). The study aimed to assess whether EQ-5D-3L would be appropriate for assessing the impact of epilepsy, treatment and potential side effects on HRQoL. A disease-specific measure, the QOLIE-31P, and other indicators such as presence and number of seizures were used as the basis of the assessment.

Convergent validity examined the correlation between EQ-5D-3L and QOLIE-31P for both overall and dimension/subscale scores, and the correlation between each measure and seizure frequency using Pearson’s (between EQ-5D-3L utility score and QOLIE-31P scores with seizure frequency) or Spearman’s rank (between EQ-5D-3L dimensions and seizure frequency) correlations. Cohen’s *d* cut-offs were used to assess the strength of correlations: ≥0.5, strong; ≥0.3 to <0.5, moderate; <0.3, weak [17]. Moderate-to-strong correlations were expected where HRQoL concepts were considered to be related including the QOLIE-31P emotional well-being and seizure worry scores with the EQ-5D-3L anxiety/depression score, and the QOLIE-31-P daily activities/social functioning and energy/fatigue scores with the EQ-5D-3L usual activities score.

Known group validity analysis assessed the ability of the EQ-5D-3L to discriminate between groups expected to differ in HRQoL at baseline, based on QOLIE-31P and other measures of severity:

- QOLIE-31P scores of 0–40, 41–50, 51–60, 61–70 and 71–100 were used to represent severity groups; the categorization reflects the QOLIE-31P total score distribution as there were no published cut-offs. This provided additional information to correlation analysis on whether EQ-5D-3L performed in a similar way across the QOLIE-31P total score range
- Presence of secondarily generalized focal seizures during baseline (yes, no) (proxy for severe epilepsy)
- Presence of seizures on the day of completing the instrument (yes, no)
- Baseline focal seizure frequency/week (<1, 1–<2, ≥2)
- Number of prior AEDs (0–1, 2–4).

Mean differences and effect sizes (ES; mean difference/pooled standard deviation [SD]) across severity subgroups were calculated for these variables. ES cut-offs were defined as: 0.2–<0.5, small; 0.5–<0.8, medium; ≥0.8, large [17]. One-way analysis of variance (ANOVA) or *t* tests were used to assess differences in EQ-5D-3L utility scores and QOLIE-31P total scores. As EQ-5D-3L utility scores were not distributed normally, they were also assessed using the nonparametric Kruskal–Wallis and Mann–Whitney tests. Statistical significance was based on *α* = 0.05. Baseline QOLIE-31P scores of patients with an EQ-5D-3L utility score of 1 (best possible health status) were also assessed to provide information on how well the EQ-5D-3L classified patients.

Pooled baseline data for patients with no missing EQ-5D-3L or QOLIE-31P data (*n* = 1095, 97% of the total sample) were used to assess convergent and known group validity.

#### Responsiveness

Responsiveness is the ability of an instrument to detect changes. In order to do so, a measure should be sensitive enough to reflect the dimensions of concern as well as the full range of severity in a given population. It should also reflect change where it has occurred.

The proportion of individuals reporting the lowest or highest scores at baseline was examined in EQ-5D-3L dimensions compared to QOLIE-31P dimensions. If the majority of individuals are at the highest score, this may indicate that the dimension is not relevant or sensitive enough particularly if other measures are sensitive enough to detect problems in the same population. Lack of sensitivity has an impact on the ability of the instrument to detect improvements while a large proportion at the lowest score makes it difficult to detect deterioration.

Mean change and standardized response means (SRM; mean change/change SD) in EQ-5D-3L utility scores and QOLIE-31P total and subscale scores from baseline to follow-up were calculated. SRMs were compared across both measures as small SRMs may reflect lack of change in the group rather than lack of responsiveness of the EQ-5D-3L. SRM cut-offs were defined as: 0.2–<0.5, small; 0.5–<0.8, medium; ≥0.8, large [17].

Responsiveness was based on change in EQ-5D-3L utility scores and QOLIE-31P total scores for: responders (≥50% reduction in focal seizure frequency at follow-up) vs non-responders; PGES (scores of 1–4 vs. 5 vs. 6 vs. 7); and CGES (scores of 1–4 vs. 5 vs. 6 vs. 7). Both PGES and CGES are reported as there may be differences in patient and investigator perception of change. One-way ANOVA or *t* tests were used to assess the magnitude of differences in the EQ-5D-3L and QOLIE-31P scores across groups.

Due to different study treatment period durations, responsiveness was assessed using individual study datasets. The analysis population comprised all patients with no missing EQ-5D-3L or QOLIE-31P data at baseline and follow-up (week 12 or week 16) (*n* = 969, 86%).

#### Mapping

Mapping between the QOLIE-31P and the EQ-5D-3L was a secondary study objective that was contingent on the psychometric analysis results. However, psychometric results indicated that mapping was not appropriate based on strength of correlations, ability to distinguish between groups with known differences and responsiveness and this was confirmed in separate mapping assessment (see Online Supplement 1).

All the validity analyses were undertaken using Stata 12.2 (StataCorp LP, College Station, TX, USA).

## Results

Overall, 1095 patients had both EQ-5D-3L and QOLIE-31P data available. Across the three studies, mean age of the population was 36.8–39.2 years, there were marginally more males than females, and the majority were Caucasian (Table 1). Median focal seizure count/28 days at baseline was 1.91–2.48 and the mean number of prior AEDs was ≈3 across the three studies. Pooled mean (SD) EQ-5D-3L utility score was 0.76 (0.23) at baseline and 0.78 (0.23) at follow-up, while pooled mean (SD) QOLIE-31P total scores were 55.6 (16.0) and 60 (16.1) at baseline and follow-up, respectively. QOLIE-31P subscale scores are given in Online Supplement 1.

**Table 1:** Baseline demographics, epilepsy characteristics, and baseline and follow-up health-related quality of life scores of patients included in the analysis

Characteristic	Brivaracetam study			
	N01252 (*N* = 355)	N01253 (*N* = 347)	N01254 (*N* = 393)	Pooled (*N* = 1095)
Age [mean (SD)]	37.5 (13.0)	39.2 (12.1)	36.8 (11.5)	37.8 (12.2)
Male, [*n* (%)]	202 (56.9)	176 (50.7)	200 (50.9)	578 (52.8)
Race [*n* (%)]				
Caucasian	280 (78.9)	248 (71.5)	232 (59.0)	760 (69.4)
Other	75 (21.2)	99 (28.5)	161 (41.0)	335 (30.6)
Baseline focal seizure frequency/28 days				
Mean (SD)	4.01 (6.5)	6.37 (12.3)	5.06 (12.4)	5.13 (3.04)
Median (IQR)	1.91 (2.3)	2.48 (4.0)	2.21 (3.2)	2.14 (3.04)
Number of prior AEDs [mean (SD)]	3.5 (2.43)	3.3 (2.53)	3.1 (2.18)	3.3 (2.38)
EQ-5D-3L [mean (SD)]				
Baseline	0.756 (0.234)	0.762 (0.226)	0.758 (0.234)	0.759 (0.232)
Follow-up	0.770 (0.241)	0.791 (0.221)	0.771 (0.229)	0.777 (0.230)
QOLIE-31P total score [mean (SD)]				
Baseline	56.3 (16.3)	54.8 (17.1)	55.6 (14.7)	55.6 (16.0)
Follow-up	59.8 (15.8)	59.6 (17.6)	60.2 (15.0)	59.9 (16.1)

*AED* antiepileptic drug, *IQR* interquartile range, *QOLIE*-*31P* Quality of Life in Epilepsy Inventory, *SD* standard deviation

### Convergent validity

No strong correlations between EQ-5D-3L dimensions and QOLIE-31P subscales were noted. At baseline, the EQ-5D-3L usual activities dimension had weak-to-moderate correlations with QOLIE-31P daily activities/social functioning (*ρ* = −0.319), emotional well-being (*ρ* = −0.316), energy/fatigue (*ρ* = −0.290), and cognitive function subscales (*ρ* = −0.286) (Table 2). EQ-5D-3L anxiety/depression had moderate correlations with emotional well-being (*ρ* = −0.455) and energy/fatigue (*ρ* = −0.334), but the correlation with the seizure worry subscale was lower than expected (*ρ* = −0.274). There were moderate correlations between QOLIE-31P overall quality of life with EQ-5D-3L usual activities (*ρ* = −0.327) and anxiety/depression (*ρ* = −0.397). Most other correlations were weak (*ρ* < –0.3), with little evidence of association between mobility, self-care, and pain/discomfort and the QOLIE-31P subscales. There were no associations between EQ-5D-3L or QOLIE-31P dimensions/subscales and seizure frequency.

**Table 2:** Convergent validity of EQ-5D-3L and QOLIE-31P using Spearman’s rank correlation at baseline (pooled data)

EQ-5D-3L dimensions	Mobility	Self-care	Usual activities	Pain/discomfort	Anxiety/depression	EQ-5D-3L utility score
QOLIE-31P subscales (*n* = 1076)						
Energy/fatigue	−0.188	−0.115	−0.290	−0.232	−0.334	0.363
Emotional well-being	−0.197	−0.132	−0.316	−0.255	−0.455	0.449
Daily activities/social functioning	−0.163	−0.151	−0.319	−0.253	−0.234	0.345
Cognitive functioning	−0.166	−0.177	−0.286	−0.238	−0.288	0.348
Medication effects	−0.156	−0.074	−0.261	−0.229	−0.205	0.285
Seizure worry	−0.106	−0.102	−0.261	−0.173	−0.274	0.280
Overall QoL	−0.233	−0.127	−0.327	−0.274	−0.397	0.424
QOLIE-31P total score	−0.234	−0.197	−0.413	−0.328	−0.427	0.496
Focal seizure frequency (*n* = 1072)	0.040 ns	0.052 ns	0.065	0.026 ns	−0.032 ns	−0.031 ns

Cohen’s cut-offs: ≥0.5, strong; ≥0.3–<0.5, moderate; <0.3, weak

*ns* not significant, *QoL* quality of life, *QOLIE*-*31P* Quality of Life in Epilepsy Inventory

The EQ-5D-3L utility score had moderate correlations with all QOLIE-31P subscales (*ρ* = 0.345–0.496) except medication effects and seizure worry, which were weakly correlated (*ρ* = 0.285 and 0.280, respectively). There was no association between EQ-5D-3L utility score and seizure frequency (*ρ* = −0.031).

### Known group validity

Mean EQ-5D-3L utility scores varied with QOLIE-31P groups, and differences across the groups were statistically significant (*p* < 0.001), with mainly small ES between groups (Table 3). The presence of secondarily generalized focal seizures (severe seizures) was associated with statistically significant lower EQ-5D-3L utility scores and QOLIE-31P scores (both *p* < 0.001). ES were small for both EQ-5D-3L (−0.21) and QOLIE-31P (−0.32). Few patients reported seizures on the day they completed the instrument (*n* = 82). There were no statistically significant differences between EQ-5D-3L and QOLIE-31P scores for patients who reported seizures versus those who did not (Table 3). Further assessment indicated there was no monotonic relationship between the number of seizures that patients reported and either measure. There was some evidence that patients with ≥5 prior AEDs had lower health status based on a statistically significant difference between EQ-5D-3L utility scores (*p* = 0.002). ES for this difference were small (−0.22), but were larger than the equivalent ES for QOLIE-31P total score (−0.11).

**Table 3:** Known group validity of EQ-5D-3L and QOLIE-31P at baseline (pooled data)

Variable	Groups	*N*	EQ-5D-3L utility score			QOLIE-31P total score		
			Mean (SD)	ES	Test statistic/*p* value	Mean (SD)	ES	Test statistic/*p* value
QOLIE-31P total score groups	0–40	195	0.575 (0.28)		*F*_4,1090_ = 73.8 *p* < 0.001			
	41–50	210	0.697 (0.22)	0.53		–	–	
	51–60	280	0.779 (0.20)	0.36		–	–	
	61–70	208	0.832 (0.16)	0.23		–	–	
	71–100	202	0.897 (0.15)	0.28		–	–	
Secondarily generalized focal seizures at baseline	No	734	0.775 (0.22)		*t*_1089_ = 3.3 *p* < 0.001	57.2 (15.4)		*t*_1089_ = 5.0 *p* < 0.001
	Yes	357	0.726 (0.26)	−0.21		52.1 (16.8)	−0.32	
Any seizure on day of completion of PRO	No	968	0.759 (0.23)		*t*_1048_ = −0.98 *p* = 0.327	55.5 (16.1)		*t*_1048_ = −1.5 *p* = 0.138
	Yes	82	0.785 (0.24)	0.11		58.3 (15.7)	0.17	
Baseline focal seizure frequency/week (log)	<1	418	0.765 (0.23)		*F*_2,1088_ = 1.16 *p* = 0.314	56.3 (14.8)		*F*_2,1088_ = 3.0 *p* = 0.050
	1–<2	485	0.748 (0.24)	−0.07		54.2 (17.1)	–0.13	
	≥2	188	0.775 (0.21)	0.12		57.1 (15.8)	0.18	
Number of prior AEDs in the past 5 years	0–1	331	0.780 (0.22)		*F*_2,1092_ = 6.12 *p* = 0.002	56.3 (16.1)		*F*_2,1092_ = 1.6 *p* = 0.202
	2–4	479	0.768 (0.22)	−0.05		55.9 (15.4)	−0.02	
	≥5	285	0.718 (0.25)	−0.22		54.1 (17.0)	−0.11	

*AED* antiepileptic drug, *ES* effect sizes, *PRO* patient-reported outcome, *QOLIE*-*31P* Quality of Life in Epilepsy Inventory, *SD* standard deviation

Cohen’s cut-offs: ≥ 0.8, large; ≥0.5–<0.8, medium; ≥0.2–<0.5, small

Across the three studies, a number of patients had full health based on the EQ-5D-3L utility scores (24.9%). However, the majority of these patients (>84%) reported less than full health in QOLIE-31P total scores and subscales (Table 4).

**Table 4:** QOLIE-31P scores for those with EQ-5D-3L utility score = 1 (pooled data)

Variable	*N*	Mean	SD	Median	Min	Max	% with less than max QOLIE score
QOLIE-31P subscales							
Energy/fatigue	273	61.6	17.7	65.0	10.0	100	98.9
Emotional well-being	273	73.6	16.0	76.0	24.0	100	95.6
Daily activities/social functioning	273	67.0	22.5	66.0	0.0	100	86.4
Cognitive functioning	273	66.2	24.0	67.8	0.0	100	89.0
Medication effects	273	67.4	23.6	69.4	0.0	100	84.2
Seizure worry	273	54.6	27.6	59.3	0.0	100	94.5
Overall QoL	273	68.9	15.3	72.5	22.5	100	96.0
QOLIE total score	273	66.4	14.1	65.8	31.1	97.6	100

*Max* maximum, *Min* minimum, *QoL* quality of life, *QOLIE*-*31P* Quality of Life in Epilepsy Inventory, *SD* standard deviation

### Responsiveness

There was little evidence at baseline of a large proportion of respondents reporting the lowest levels in EQ-5D-3L and QOLIE-31P dimensions/subscales. However, all five EQ-5D-3L dimensions had a large proportion reporting no problems (mobility: 83, 85, 81; self-care: 92, 93, 90%; usual activities: 64, 62, 62%; pain/discomfort: 52, 49, 52%; anxiety/depression: 43, 48, 43%) in the N01252, N01253 and N01254 studies, respectively. This is consistent with the fact that mobility and self-care were expected to be less problematic in this population. The QOLIE-31P subscales did not have equivalent large proportions reporting no problems except for medication effects in trial N01254 which was at 11%.

Mean changes in EQ-5D-3L dimensions were not statistically significant with small SRMs (SRM ≈ 0.1 in all three trials). Mean changes in QOLIE-31P subscales were also small, but they were statistically significant (*p* < 0.05) and larger than EQ-5D-3L changes (SRM = 0.1–0.4)) except for medication effects (ES ≤ 0.1). The largest SRM in the QOLIE-31P was in seizure worry in studies N01252 and N01254 (SRM = 0.3 and 0.4, respectively). This indicated minor improvements in health status and HRQoL over time based on the disease-specific measure.

For responsiveness based on ≥50% reduction in focal seizures, changes in EQ-5D-3L utility scores were higher in responders vs non-responders in studies N01253 (ES = 0.41) and N01254 (ES = 0.11), but the difference was statistically significant only in study N01253 (*p* = 0.002) (Table 5). In contrast, QOLIE-31P total score change was statistically significantly higher in responders in all three studies.

**Table 5:** Responsiveness based on response to treatment status and clinician/patient evaluation of change at follow-up

Variable	Groups	*N*	EQ-5D-3L utility score			QOLIE-31P total score		
			Mean change (SD)	ES	ANOVA/*t* test (*p* value)	Mean change (SD)	ES	ANOVA/*t* test (*p* value)
N01252								
Response to treatment (≥50% reduction in focal seizure frequency)	Non-responders	226	0.018 (0.24)		*t*_315_ = 0.07 *p* = 0.947	2.01 (12.4)		*t*_315_ = −3.7 *p* < 0.001
	Responders	91	0.016 (0.21)	−0.01		7.94 (14.5)	0.45	
PGES	1–4	96	0.016 (0.22)		*F*_3,308_ = 0.09 *p* = 0.966	−1.07 (9.6)		*F*_3,308_ = 9.25 *p* < 0.001
	5	87	0.027 (0.21)	0.05		3.67 (10.9)	0.36	
	6	73	0.009 (0.28)	−0.08		6.58 (14.6)	0.22	
	7	56	0.020 (0.18)	0.05		9.13 (16.3)	0.19	
CGES	1–4	108	0.051 (0.21)		*F*_3,312_ = 1.7 *p* = 0.167	0.61 (12.1)		*F*_3,312_ = 5.6 *p* < 0.001
	5	95	0.022 (0.22)	−0.13		3.57 (11.2)	0.23	
	6	76	−0.023 (0.25)	−0.20		5.96 (13.4)	0.18	
	7	37	0.005 (0.20)	0.12		9.73 (16.7)	0.29	
N01253								
Response (≥50% reduction in focal seizure frequency)	Non-responders	234	0.000 (0.25)		*t*_303_ = −3.1 *p* = 0.002	3.46 (12.6)		*t*_303_ = −3.2 *p* = 0.001
	Responders	71	0.103 (0.25)	0.41		9.71 (18.7)	0.43	
PGES	1–4	97	−0.003 (0.21)		*F*_3,297_ = 1.9 *p* = 0.123	−0.76 (11.5)		*F*_3,297_ = 9.2 *p* < 0.001
	5	68	0.005 (0.33)	0.03		5.51 (11.6)	0.44	
	6	77	0.020 (0.19)	0.06		6.99 (13.3)	0.10	
	7	59	0.092 (0.28)	0.28		10.34 (19.1)	0.23	
CGES	1–4	113	−0.004 (0.21)		*F*_3,297_ = 2.8 *p* = 0.043	0.46 (12.9)		*F*_3,297_ = 9.9 *p* < 0.001
	5	83	−0.004 (0.30)	0.00		4.91 (11.7)	0.31	
	6	65	0.054 (0.22)	0.23		6.93 (14.0)	0.14	
	7	40	0.111 (0.28)	0.23		13.64 (18.4)	0.47	
N01254								
Response (≥50% reduction in focal seizure frequency)	Non-responders	244	0.010 (0.23)		*t*_342_ = −0.92 *p* = 0.356	3.16 (12.0)		*t*_342_ = −4.2 *p* < 0.001
	Responders	100	0.036 (0.26)	0.11		9.38 (14.0)	0.48	
PGES	1–4	111	−0.021 (0.21)		*F*_3,335_ = 3.6 *p* = 0.015	−0.20 (10.6)		*F*_3,335_ = 19.3 *p* < 0.001
	5	92	0.048 (0.23)	0.28		3.29 (12.0)	0.28	
	6	88	−0.011 (0.24)	−0.24		8.33 (12.0)	0.40	
	7	48	0.096 (0.33)	0.44		13.85 (13.3)	0.43	
CGES	1–4	128	−0.016 (0.20)		*F*_3,341_ = 1.9 *p* = 0.138	−1.12 (10.6)		*F*_3,341_ = 21.7 *p* < 0.001
	5	86	0.010 (0.27)	0.11		5.12 (13.6)	0.48	
	6	91	0.059 (0.27)	0.20		11.36 (11.9)	0.48	
	7	40	0.039 (0.23)	−0.08		9.17 (11.8)	−0.17	

PGES and CGES categories: 1–4, marked worsening to no change; 5, slight improvement; 6, moderate improvement; 7, marked improvement

Cohen’s cut-offs: ≥0.8, large; ≥0.5–<0.8, medium; ≥0.2–<0.5, small

*ANOVA* analysis of variance, *CGES* Clinician’s Global Evaluation Scale, *ES* effect sizes, *PGES* Patient’s Global Evaluation Scale, *QOLIE*-*31P* Quality of Life in Epilepsy Inventory, *SD* standard deviation

Assessment of response based on PGES and CGES showed mostly no statistically significant differences between groups with small ES in EQ-5D-3L utility scores (Table 5). EQ-5D-3L utility scores differed based on CGES groups for study N01253 and for PGES groups for study N01254, but the latter was not a monotonic relationship. In contrast, QOLIE-31P total score change had a linear association with the statistically significant improvements reported in PGES and CGES (*p* < 0.001), except for CGES in study N01254.

## Discussion

To assess the psychometric properties of the EQ-5D-3L in patients with uncontrolled focal seizures, we used data from three large Phase III, randomized, placebo-controlled studies of brivaracetam, which is approved as adjunctive therapy for focal seizures in adults with epilepsy. Epilepsy-specific measures including the QOLIE-31P were used as proxies for severity in convergent and known group analysis. The responsiveness of EQ-5D-3L and QOLIE-31P was assessed based on their ability to detect differences in treatment outcome groups.

Despite differences in the focus of the measures (generic vs disease-specific), some association between measures was expected. Correlation analyses confirmed some association between similar dimensions/subscales of each instrument, although generally it was weak. Contrary to expectations, the EQ-5D-3L usual activities and the QOLIE-31P energy/fatigue and daily activities/social functioning subscales were only weakly correlated. Similarly, only a weak correlation was observed between EQ-5D-3L anxiety/depression and QOLIE-31P seizure worry. An earlier study reported that some patients with epilepsy had difficulty answering the anxiety/depression dimension of the EQ-5D-3L as they did not consider themselves to be depressed [18]. Therefore, patients may not have considered seizure worry when completing the more general anxiety/depression questions of the EQ-5D-3L. Dimensions relating to mobility and self-care had little association with QOLIE-31P subscales, and there were mainly weak correlations between the pain dimension and QOLIE-31P subscales. This might be expected, as these aspects of HRQoL may not be impaired in patients with epilepsy. EQ-5D-3L utility scores had weak-to-moderate correlations with the QOLIE-31P subscales. Baseline seizure frequency was neither correlated with the EQ-5D-3L dimensions or utility scores, nor with QOLIE-31P subscale scores.

The EQ-5D-3L was able to reflect differences in groups based on the QOLIE-31P total score. Neither EQ-5D-3L nor QOLIE-31P scores reflected differences in baseline number of seizures. Poor association between seizure frequency and HRQoL may be due to the severity or timing of seizures experienced. The episodic nature of epilepsy means that seizure-free periods can be associated with good HRQoL which decreases following a seizure [2]. Furthermore, EQ-5D-3L asks patients about their health on the day of assessment, whereas QOLIE-31P covers the past 4 weeks and attempts to get a reading of ‘average’ HRQoL. The presence of seizures on the day of questionnaire completion was not negatively associated with HRQoL in either measure; however, it was unknown whether seizures occurred before or after questionnaire completion. The lack of association between seizure frequency and HRQoL may also be because large gains in HRQoL are only achieved with seizure freedom [19], and this was achieved by relatively few patients. However, there was evidence to suggest that seizure severity may impact on HRQoL; patients with secondarily generalized seizures, a proxy for more severe seizures, had lower health status/HRQoL in both EQ-5D-3L and QOLIE-31P, although the ES for EQ-5D-3L were smaller. This observation is consistent with several previous studies which found negative associations between seizure severity and HRQoL [20].

EQ-5D-3L had large proportions reporting no problems in the dimensions particularly in the mobility and self-care dimensions (80–90%), which was not unexpected as patients were not expected to have problems ‘walking about’ or ‘washing and dressing’ themselves. In terms of the overall EQ-5D-3L utility score, 24.9% were in full health. The QOLIE-31P did not have comparable proportions without problems. The vast majority of respondents who reported a score of 1 in EQ-5D-3L reported scores lower than 100 (best functioning) in the QOLIE-31P subscales. This indicated that EQ-5D-3L dimensions were not relevant or sensitive enough to assess the impact of epilepsy-specific symptoms in this population. Where concepts do overlap between the measures, QOLIE-31P has more items and so may be able to capture these effects better than the single items of the EQ-5D-3L. Langfitt et al. [10] found that the SF-6D, which also has more items per dimension and similar dimensions (social, role functioning, energy, and emotional well-being), performed better than the EQ-5D-3L.

In terms of responsiveness, the EQ-5D-3L and QOLIE-31P dimension/subscale scores all showed positive change over the trial period, but only QOLIE-31P subscales had statistically significant changes. The SRMs were smaller for EQ-5D-3L utility score than for QOLIE-31P (0.1 vs. 0.1–0.4), indicating that small changes in the population were captured by some of the QOLIE-31P subscales but not by the EQ-5D-3L dimensions. Responsiveness based on 50% seizure frequency reduction indicated small ES in EQ-5D-3L utility and QOLIE-31P total scores (−0.01 vs. 0.45). The efficacy gain in terms of seizure frequency in this population is more modest than in less refractory patients and, as noted, few patients achieve seizure freedom; as such, it may be more difficult to show improvement in HRQoL [19, 21]. In addition, meaningful changes in seizure frequency, coping and lifestyle as a consequence of treatment efficacy may not be reflected in HRQoL outcomes in studies of short duration [22]. In contrast to the QOLIE-31P, change in EQ-5D-3L utility scores was largely not associated with patient and clinician evaluations of improvement. These results suggest that even if the outcome achieved in this population was modest, the QOLIE-31P detected some improvements that the EQ-5D-3L was not sensitive enough to reflect.

The psychometric analyses indicated that QOLIE-31P would be poor predictors of EQ-5D-3L due to the lack of sufficient overlap between measures evidenced by lower sensitivity of the EQ-5D-3L. This highlights the importance of assessing that generic preference-based measures are appropriate in the population of interest in terms of psychometric properties before carrying out mapping analysis. However, the applicability of the generic measure in the patient population concerned is not always reported in mapping studies.

Overall, the results suggest that although there is some association between the EQ-5D-3L and the QOLIE-31P, this is not sufficient to capture changes over time to the same degree, as the latter measure includes epilepsy-specific concepts such as seizure worry. The existing disease-specific measures, such as the QOLIE-31P, could be converted into a preference-based measure, which could then be applied to existing datasets without utility values. Alternatively, other more broad generic HRQoL measures could be used in mapping studies (e.g. the SF-36 and thus the SF-6D). Finally, utility values could be generated from an existing epilepsy-specific QALY measure, such as the Quality of Life in Newly Diagnosed Epilepsy Instrument 6 dimensions (NEWQOL-6D), which is derived from the NEWQOL [23, 24]. Values for NEWQOL-6D health states were found to be similar in patients and the general population, suggesting that using general population utility weights to estimate QALYs is appropriate and generally represents patient preferences [24]. If the measure proves to be psychometrically valid in patients with uncontrolled focal seizures, the NEWQOL-6D could be used as an alternative to generate QALYs [24].

A number of studies have assessed the performance of EQ-5D-3L in populations with epilepsy [10, 25, 26]. Overall, results support the findings of this study in that some EQ-5D-3L dimensions may be relevant to a population with uncontrolled focal seizures. However, outcomes such as seizure control may not be as closely associated with the EQ-5D-3L. Differences in levels of seizure severity and interventions make it difficult to compare these studies directly. One study, which assessed the relationship between seizure frequency and preference-based HRQoL, found that in patients with recurrent seizures, seizure frequency was not monotonically related to preference-based HRQoL, with substantial overlap across different seizure frequency categories, thereby mirroring some of the findings in this study [27].

The analyses presented in this study provide important information on the performance of EQ-5D-3L in a patient population with uncontrolled focal seizures; however, there are a number of limitations. The studies used in the analysis were designed to assess the efficacy, safety and tolerability of adjunctive brivaracetam; assessment of HRQoL was an exploratory objective, and this may have impacted on the analysis of HRQoL. The study populations were based on clinical (e.g. seizure frequency) rather than HRQoL criteria; therefore, their HRQoL data may not be applicable to the overall population of patients with uncontrolled focal seizures. Furthermore, the nature of the instruments themselves may affect results as the QOLIE-31P covers the previous 4 weeks, whereas EQ-5D-3L focuses on a single day, which may exclude typical HRQoL effects that occur over a period of time.

In summary, while some EQ-5D-3L dimensions overlapped with similar concepts in the disease-specific QOLIE-31P, the content of the measure was unable to capture self-reported epilepsy-specific concerns or to reflect change over time. Given the lack of correlation and joint responsiveness between the measures, using the EQ-5D-3L for cost-effectiveness analysis including from mapping is not recommended. A disease-specific preference-based measure may offer an alternative.

## Electronic supplementary material

Below is the link to the electronic supplementary material.

Supplementary material 1 (DOCX 1946 kb)
