# Supplementary material for: Sensitivity and responsiveness of the EQ-5D-3L in patients with uncontrolled focal seizures: an analysis of Phase III trials of adjunctive brivaracetam
Source: Qual Life Res. 2016 Dec 21;26(3):749–59. doi: 10.1007/s11136-016-1483-3 (PMC5309305; doi:10.1007/s11136-016-1483-3)
Supplement: Supplementary file 1 — Supplementary material 1 (DOCX 1946 kb) [file 11136_2016_1483_MOESM1_ESM.docx]

**SUPPLEMENTARY INFORMATION FOR QUALITY OF LIFE RESEARCH**

**Mapping between QOLIE-31P and EQ-5D-3L**

Clara Mukuria,^1^ Tracey Young,^1^ Anju Keetharuth,^1^ Simon Borghs,^2^ John Brazier^1^

^1^School of Health and Related Research, University of Sheffield, Sheffield, UK;

^2^UCB Pharma, Slough, UK

**Methods**

***Target measure***

The target measure was the preference-based utility score from the EQ-5D-3L

***Source measures***

The source measure for the statistical regression analyses was the disease-specific Quality of Life in Epilepsy Inventory (QOLIE-31P) instrument. Following standard mapping approach used in the literature (Longworth and Rowen 2013), different specifications were used including using the QOLIE-31P domains and items in order to identify which variables were best for predicting EQ-5D-3L utility scores. Other variables assessed for inclusion in the models, included age, gender, focus localisation, seizure frequency, seizure type and epilepsy syndrome.

***Statistical methodology***

All analyses were carried out on the combined datasets from the three studies. The estimation dataset used for initial testing of the regression models comprised baseline data for the three clinical studies with no missing EQ-5D-3L or QOLIE-31P data. If the models performed well, external validation was planned with the follow-up data from the studies.

Data from all three studies had ≤5% of missing individual follow-up data for EQ-5D-3L or QOLIE-31P. No imputation of missing data was made apart from the algorithm which formed part of the QOLIE-31P.

***Regression models***

Three different regression methods were used for mapping QOLIE-31P onto EQ-5D-3L: ordinary least squares (OLS) regression, Tobit modelling and two-part modelling (TPM).

OLS regressions explore relationships between explanatory variables and the dependent variable. A simple linear model was used of the form:

*DV* = β_0_ + β_1_**x*_1_ + β_2_**x*_2_ +….. + β_n_**x*_n_ + ε

where *DV* represents the EQ-5D-3L preference-based utility score, *x*_n_ represents the vector of explanatory variables, β represents the vector of weights for the explanatory variables and ε represents the residual or error term. EQ-5D-3L utility scores are bounded by limits (in this case, -0.594 to 1), which are not accounted for by OLS; therefore, the predicted values could be >1 or <-0.594 under OLS models.

Due to the distribution of EQ-5D-3L preference-based utility scores, the second approach used Tobit modelling (Tobin, 1958). Tobit models can be used to ’censor’ the upper and lower limits so predictions are not beyond the credible range of the underlying variable. To estimate predicted EQ-5D-3L values using Tobit coefficients, a linear predictor was fitted and this was adjusted so that scores were unable to go above 1 or below -0.594, i.e., the expected value of yi* (E(yi*)) was estimated:

yi* = Maximum{a, Minimum(yi, b)}

where a is the lower limit, b is the upper limit and yi* is estimated from the conditional expected value of EQ-5D-3L predictions xb – sigma*pdf{(1-xb)/sigma}*p[(1/xb)/sigma] and the probability of being within the limits of the distribution.

The EQ-5D-3L utility scores in the three trials did not cover the full range to the lower limit; therefore, only an upper limit was imposed on the Tobit model.

TPM also deals with the distribution of the EQ-5D-3L utility score and uses a combination of two different model types to predict different parts of the distribution of the data. A mathematically defined version of the TPM was used, which fits a logistic regression to estimate those in perfect health (Yes/No) and a truncated OLS model to predict EQ-5D-3L utility score for those not in perfect health. The truncated OLS model score cannot exceed 0.99 (Huang et al, 2008). EQ-5D-3L utility scores were estimated as follows:

Expected (EQ-5D-3L) = 1*Probability(PH) + (EQ-5D-3L score if not PH*(1 – Probability(PH))

where PH is perfect health.

***Assessing mapping performance and validation***

The regression results were assessed using several criteria. Predictive ability of the models was assessed using the mean absolute error (MAE; mean of the absolute difference between individual estimated and observed utilities). The distribution of error across the full EQ-5D-3L range was examined to identify systematic bias in the predictions (e.g., models obtained using OLS regressions tend to under-predict at the top and over-predict at the bottom of the EQ-5D-3L utility score ranges). Prediction accuracy by severity groups was also assessed.

To select the best model to use, predicted values were also assessed in terms of the MAE across the full index, the range in predicted values, the reproducibility of the EQ-5D-3L values across different severity states and the accuracy of the predicted mean and standard error. Based on the follow-up data and subject to the performance of the models in the estimation sample, external validation was planned.

To assess that the models were meeting *a priori* expectations based on examining the QOLIE-31P data, a number of further tests were conducted. The direction and statistical significance of the coefficients for the explanatory variable were checked to determine if they were in the expected direction. The explanatory power of the models was assessed using R^2^ and adjusted R^2^ (or Pseudo R^2^ where appropriate). Goodness of fit was assessed using the Akaike information criteria (AIC) and the Bayesian information criteria (BIC). To test for heteroscedasticity White’s test was used. Influential observations and outliers were investigated and sensitivity analysis was undertaken using Cook’s Distance (Cook, 1977) to measure influence, and models were re-estimated without the influential observations to assess the impact of removing them. The Ramsey Regression Equation Specification Error Test (RESET) was used with a 10% significance level in the linear models obtained using OLS to identify omitted variables.

**Mapping analysis results**

The EQ-5D-3L utility scores did not cover the full possible range of scores (-0.594 to 1) at baseline, with a mean utility score of 0.757 (standard deviation: 0.235). Data were negatively skewed; median utility score (0.796) was greater than the mean. Few patients had scores <1 while 25% of patients had scores of 1 and most patients reported no or some problems in the five dimensions, with very few reporting extreme problems. There were no statistically significant differences in EQ-5D-3L utility scores between subgroups of other demographic variables considered for source measures, except for severe vs non-severe seizures (p<0.001).

**Regression results**

As would be expected, OLS models predicted the mean EQ-5D-3L utility scores but was poor at predicting across the range of EQ-5D-3L utility scores (Figure 1). The best minimum predicted EQ-5D-3L utility score was 0.2135 compared with an observed minimum score of -0.2390; the best maximum predicted score is 0.9406 compared with an observed maximum of 1. Overall MAEs ranged from 0.136 to 0.148 but MAEs were very large at the severe end of the EQ-5D-3L scale (>0.4) indicating poor performance. The models tended to over-predict for patients with severe seizures and under-predict for those not classified as severe.

Figure 1: Observed and predicted mean EQ-5D-3L scores – OLS


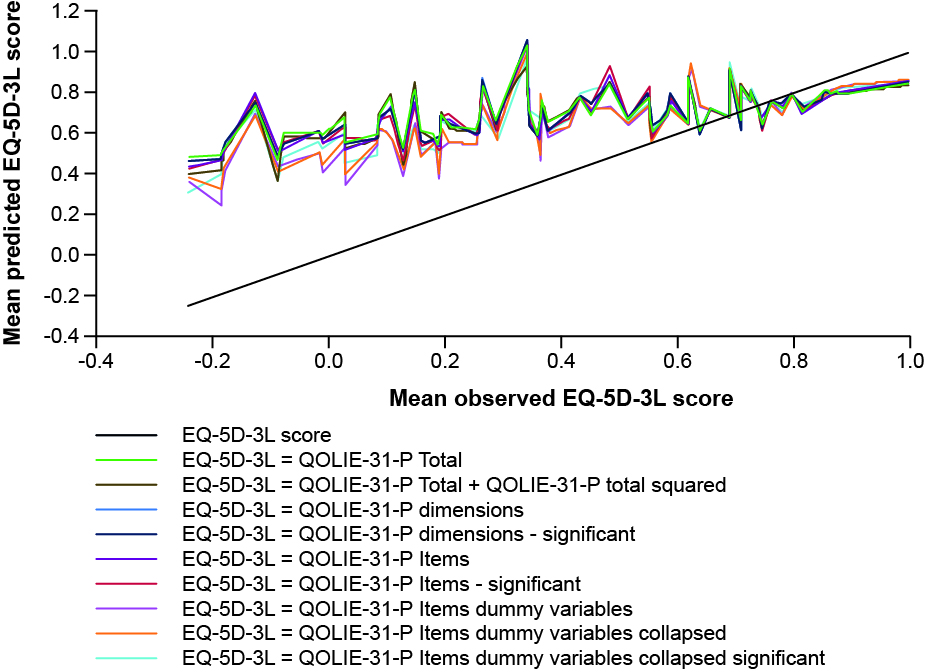


*OLS* ordinary least squares*; QOLIE-31P* Quality of Life in Epilepsy Inventory

Tobit models were also able to predict mean EQ-5D-3L utility scores but performed poorly across the range of scores (Figure 2). They over-predicted the minimum scores, with the best predicted minimum EQ-5D-3L utility score of 0.2091 compared with an observed score of -0.239. As Tobit models were ‘censored’ to the plausible range of the EQ-5D-3L utility scores, there were no values above 1. Overall MAEs ranged between 0.135 and 0.148 but were still large at the lower end (>0.4).

Figure 2: Observed and predicted EQ-5D-3L scores – Tobit


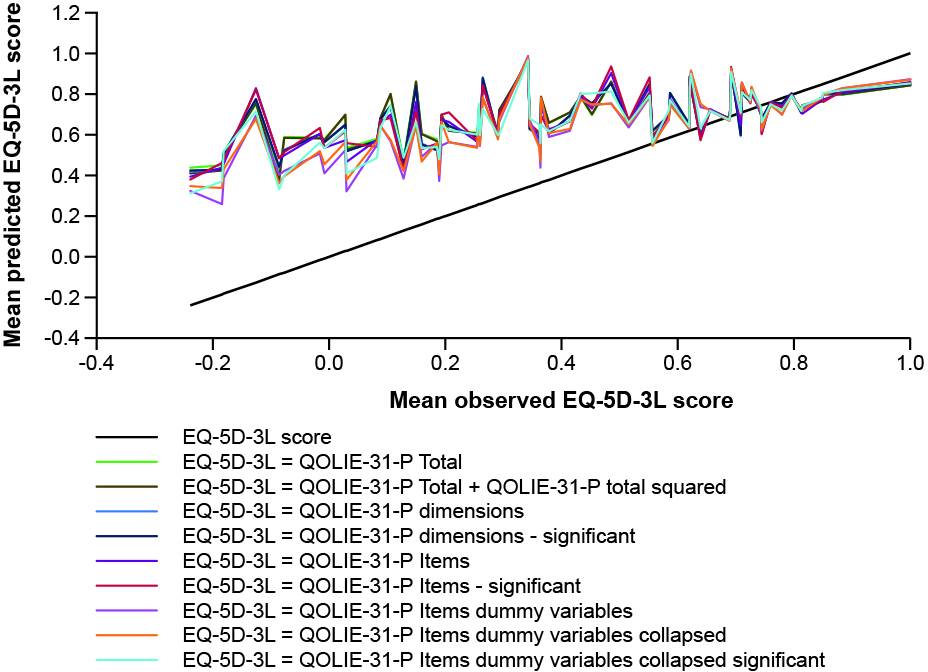


*QOLIE-31P* Quality of Life in Epilepsy Inventory

Several models using QOLIE-31P items could not be analysed for TPM due to the small proportion of patients with perfect health (25%). Compared with the OLS and Tobit regressions, the TPM regressions over-predicted the mean EQ-5D-3L utility score. The best predicted mean score was 0.8074 compared with an observed mean score of 0.7568. The best predicted minimum EQ-5D-3L utility score was 0.1569 compared with 0.2135 for OLS and 0.2091 for Tobit regressions. However, this was still above the observed minimum score of -0.239. TPM regressions also offered some improvements at the top of the scale with the best predicted maximum score of 0.9977. Overall MAEs ranged from 0.140 to 0.147 but were as large as 0.51 at the lower end of the EQ-5D-3L scale (Figure 3).

Figure 3: Observed and predicted EQ-5D-3L scores – TPM


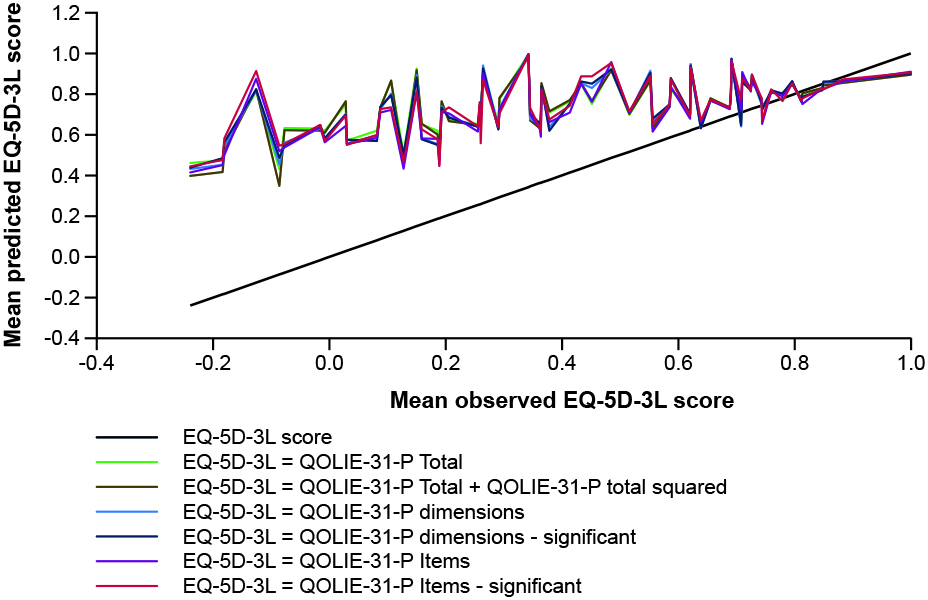
*QOLIE-31P* Quality of Life in Epilepsy Inventory; TPM two-part modelling

Given the poor performance of these regressions in the estimation sample on which they were modelled, external validation of these models on follow-up data was not undertaken. The errors identified in the estimation sample were likely to be larger in a different sample.

**Conclusion**

The mapping functions perform poorly and this is mainly due to the weak overlap between EQ-5D and QOLIE-31P. It is therefore recommended that mapping is not undertaken between these measures.

**References**

Cook RD. Detection of influential observation in linear regression. Technometrics 1977; 19: 15-18.

Huang IC, Frangakis C, Atkinson MJ *et al.* Addressing ceiling effects in health status measures: a comparison of techniques applied to measures for people with HIV disease. Health Serv Res 2008; 43: 327-39.

Longworth L, and Rowen D. "Mapping to obtain EQ-5D utility values for use in NICE health technology assessments." *Value in Health* 16.1 (2013): 202-210

Tobin J. Estimation of relationships for limited dependent variables. Econometrica 1958; 29: 24-36.

**Supplement Table: Descriptive statistics for QOLIE-31P (baseline [BL] and follow-up [FUP])**

|  |  | **n** | **Mean** | **SD** | **Median** | **Min** | **Max** | **Completion %** |
| --- | --- | --- | --- | --- | --- | --- | --- | --- |
| ***N01252*** |  |  |  |  |  |  |  |  |
| **QOLIE-31P** |  |  |  |  |  |  |  |  |
| Energy/Fatigue | BL | 355 | 52.8 | 18 | 55 | 0 | 100 | 98.1 |
|  | FUP | 317 | 56 | 18.5 | 55 | 10 | 100 | 90.8 |
| Emotional well-being | BL | 355 | 61.1 | 18.7 | 60 | 0 | 100 | 98.9 |
|  | FUP | 317 | 63.8 | 17.4 | 64 | 20 | 100 | 91.3 |
| Daily Activities/ | BL | 355 | 57.1 | 22.2 | 57 | 0 | 100 | 99.5 |
| Social Functioning | FUP | 317 | 59.8 | 22.1 | 57.5 | 0 | 100 | 91.0 |
| Cognitive Functioning | BL | 355 | 56.5 | 24.6 | 56.1 | 0 | 100 | 99.5 |
|  | FUP | 317 | 59.5 | 23.1 | 59.3 | 0 | 100 | 91.0 |
| Medication Effects | BL | 355 | 57.8 | 27 | 61.1 | 0 | 100 | 99.2 |
|  | FUP | 317 | 60.1 | 24.6 | 61.1 | 0 | 100 | 91.3 |
| Seizure Worry | BL | 355 | 46.2 | 26.9 | 47 | 0 | 100 | 99.5 |
|  | FUP | 317 | 54.1 | 25.1 | 54.3 | 0 | 100 | 91.3 |
| Overall QoL | BL | 355 | 58.2 | 18 | 55 | 0 | 100 | 99.5 |
|  | FUP | 317 | 62.8 | 17 | 60 | 5 | 100 | 91.3 |
| ***N01253*** |  |  |  |  |  |  |  |  |
| QOLIE-31P |  |  |  |  |  |  |  |  |
| Energy/Fatigue | BL | 347 | 54.3 | 20.5 | 55 | 0 | 100 | 99.4 |
|  | FUP | 305 | 58.7 | 21.1 | 60 | 0 | 100 | 86.5 |
| Emotional well-being | BL | 347 | 64.4 | 19.8 | 64 | 12 | 100 | 99.4 |
|  | FUP | 305 | 67.3 | 20.3 | 68 | 4 | 100 | 87.0 |
| Daily Activities/ | BL | 347 | 51.2 | 25.6 | 51 | 0 | 100 | 100 |
| Social Functioning | FUP | 305 | 56.7 | 25.1 | 57 | 0 | 100 | 87.6 |
| Cognitive Functioning | BL | 347 | 52.1 | 26.1 | 52.2 | 0 | 100 | 99.4 |
|  | FUP | 305 | 57.6 | 25.3 | 59.4 | 0 | 100 | 87.6 |
| Medication Effects | BL | 347 | 56.3 | 28.1 | 55.6 | 0 | 100 | 99.7 |
|  | FUP | 305 | 58.5 | 28.3 | 61.1 | 0 | 100 | 87.6 |
| Seizure Worry | BL | 347 | 42.1 | 28.7 | 40.3 | 0 | 100 | 99.7 |
|  | FUP | 305 | 48.9 | 28.4 | 49.3 | 0 | 100 | 87.6 |
| Overall QoL | BL | 347 | 62.5 | 19 | 65 | 0 | 100 | 100 |
|  | FUP | 305 | 66.8 | 17.2 | 67.5 | 10 | 100 | 86.2 |
| ***N01254*** |  |  |  |  |  |  |  |  |
| QOLIE-31P |  |  |  |  |  |  |  |  |
| Energy/Fatigue | BL | 393 | 50.1 | 19.2 | 50 | 0 | 95 | 99.5 |
|  | FUP | 347 | 54.2 | 18.7 | 55 | 0 | 100 | 92.0 |
| Emotional well-being | BL | 393 | 60 | 18 | 60 | 8 | 100 | 100 |
|  | FUP | 347 | 63.2 | 17.4 | 64 | 12 | 100 | 91.8 |
| Daily Activities/ | BL | 393 | 57.4 | 21.5 | 57 | 0 | 100 | 100 |
| Social Functioning | FUP | 347 | 61.4 | 21.5 | 60 | 0 | 100 | 92.5 |
| Cognitive Functioning | BL | 393 | 57.4 | 23 | 58.1 | 0 | 100 | 99.5 |
|  | FUP | 347 | 62.3 | 21.5 | 62.8 | 3.33 | 100 | 92.8 |
| Medication Effects | BL | 393 | 58.9 | 25.3 | 61.1 | 0 | 100 | 99.5 |
|  | FUP | 347 | 60.7 | 23.9 | 61.1 | 0 | 100 | 92.8 |
| Seizure Worry | BL | 393 | 44 | 26.3 | 45.3 | 0 | 100 | 99.8 |
|  | FUP | 347 | 53.5 | 25.4 | 53.7 | 0 | 100 | 92.8 |
| Overall QoL | BL | 393 | 55.2 | 17.3 | 55 | 12.5 | 100 | 100 |
|  | FUP | 347 | 59.6 | 16.6 | 55 | 0 | 100 | 92.5 |

*BL* baseline; *FUP* follow-up; *QOLIE-31P* Quality of Life in Epilepsy Inventory; *SD* standard deviation
